# The Other Side of the Stethoscope: Clinician‐Facing Deepfakes and the Symmetry of Synthetic Deception

**DOI:** 10.1111/jep.70548

**Published:** 2026-08-02

**Authors:** George Rugare Chingarande

**Affiliations:** ^1^ Stellenbosch University Faculty of Medicine and Health Sciences, Division of Medical Ethics and Law Cape Town South Africa

**Keywords:** automation bias, clinical epistemology, deepfakes, diagnostic error, epistemic trust, patient safety, precautionary principle, synthetic media

## Abstract

**Background:**

Clinical ethics scholarship on deepfakes has focused primarily on patients as targets of synthetic deception, in which fabricated audiovisual material alters patient beliefs about clinicians and care. This focus neglects the reverse epistemic vulnerability: clinicians themselves as recipients of synthetic, adversarially generated clinical information.

**Objective:**

To develop a conceptual analysis of clinician‐facing deepfakes and their implications for clinical epistemology, diagnostic reasoning, and institutional trust.

**Methods:**

Normative and conceptual analysis drawing on virtue epistemology, philosophy of testimony, diagnostic error theory, automation bias research, and socio‐technical systems theory.

**Results:**

Clinician‐facing deepfakes constitute a distinct epistemic risk category producing three primary harms: (1) diagnostic error cascades, (2) testimonial contamination of clinical knowledge transmission, and (3) institutional epistemic fragility. We introduce the concept of epistemic role reversal, defined as the systematic decoupling of perceptual reliability from epistemic justification under adversarially generated clinical inputs, resulting in the collapse of expert perceptual advantage in bounded domains.

**Conclusions:**

Clinical ethics must address both sides of the epistemic encounter in medicine. Clinician‐facing deepfakes are not merely a symmetrical extension of patient‐facing deception but a structurally distinct class of epistemic threat. While empirical prevalence remains unknown, evidence from adjacent literatures supports their plausibility. A precautionary framework of clinical epistemic hygiene is therefore warranted, combining institutional verification infrastructure, workflow‐level safeguards, and professional epistemic norms adapted to adversarial information environments.

## 1 Introduction: The Missing Symmetry

In 2025, Tordjman et al. evaluated 264 synthetic chest X‐rays generated using generative adversarial methods. Seventeen experienced radiologists across twelve hospitals identified only 41% of synthetic images without prompting, and 75% after disclosure that synthetic images were present. Even under awareness conditions, a substantial proportion of synthetic images remained indistinguishable from authentic imaging data [1].

This finding is clinically significant not because radiologists are uniquely vulnerable, but because it demonstrates a deeper epistemic condition: expert perception in medicine depends on environments in which the authenticity of inputs is normally stable and non‐adversarial.

Clinical deepfake ethics has, to date, focused primarily on patient vulnerability. Patients may encounter fabricated videos of clinicians, misattribute authority, and make altered treatment decisions. Existing work has accordingly emphasized patient epistemic protection, content verification, and clinician communication strategies.

However, this framing is incomplete. It assumes a one‐directional vulnerability structure in clinical epistemology: clinicians as epistemic authorities, patients as epistemic recipients. Yet modern clinical environments are increasingly characterized by bidirectional epistemic exposure to synthetic media.

Clinicians now encounter potentially synthetic inputs across multiple domains: diagnostic imaging (radiology, pathology, dermatology), patient‐generated audiovisual material, biomedical literature and preprints, and digitally mediated consultation data. Each of these channels is susceptible, in principle, to synthetic generation or manipulation.

This paper develops three claims:

1. Clinicians are structurally vulnerable to synthetic deception due to **epistemic dependence**, not incompetence.
2. Clinician‐facing deepfakes generate **three distinct harms** not reducible to patient deception.
3. These harms require a new conceptual framework: **epistemic role reversal**.

*Roadmap*: The paper proceeds as follows. Section Objective, 2 explains why clinicians are structurally vulnerable to deception—not because they are incompetent, but because of epistemic dependence, perceptual trust heuristics, workflow constraints, and the limits of expertise. Section Methods, 3 identifies three distinct harms of clinician‐facing deepfakes and includes a dedicated subsection explaining why synthetic deception is ethically distinct from ordinary error and misinformation. Section Results, 4 introduces the concept of epistemic role reversal. Section Conclusions, 5 develops a precautionary framework of clinical epistemic hygiene. Section 6 articulates virtues for clinicians in adversarial epistemic environments. Section 7 addresses objections. Section 8 states limitations. Section 9 concludes.

*A note on scope and empirical status*: This paper does not claim that clinician‐facing deepfakes are currently widespread in clinical systems. Rather, it argues that (i) the technical feasibility of such systems is established, (ii) adjacent literatures demonstrate relevant vulnerabilities in clinical reasoning systems, and (iii) the resulting risk profile justifies precautionary institutional preparation. Absence of confirmed prevalence should be interpreted cautiously in adversarial domains where detection capacity may lag behind capability. A related but distinct category—patients using deepfakes to deceive clinicians, such as fabricated symptom videos for secondary gain—raises different questions of patient motivation and clinician distrust that merit separate treatment.

## 2 Why Clinicians Are Vulnerable (Not Incompetent)

Clinician vulnerability arises from the structure of clinical epistemology itself. This section explains why clinicians are specifically vulnerable to deception—not because they are incompetent or generally vulnerable, but because the structure of clinical reasoning creates dependencies that can be exploited. The claim is not that clinicians are vulnerable to all forms of error, but that they are *structurally vulnerable to deception* because they must rely on the authenticity of clinical inputs to make judgments.

### 2.1 Epistemic Dependence in Clinical Systems

Clinical medicine is fundamentally dependent on distributed epistemic systems. Radiologists interpret images generated by complex imaging chains; laboratory physicians rely on automated analytic systems; clinicians depend on research synthesized and validated elsewhere [2, 3].

This dependence is epistemically rational under conditions of background reliability. It enables scale, efficiency, and specialization. However, it also creates a structural entry point for adversarial manipulation: clinicians rarely have direct access to the provenance of individual data artifacts. The delegation of epistemic responsibility to systems, tools, and other professionals is essential for clinical efficiency, but it simultaneously creates vulnerabilities when those delegated sources cannot be trusted.

### 2.2 Perceptual Trust and Cognitive Economy

Human cognition relies on **perceptual trust heuristics**: what appears visually or instrumentally coherent is treated as prima facie real [4, 5]. This is not a cognitive flaw but an efficiency requirement—the brain cannot verify every perceptual input in real time. However, synthetic media exploit this property by preserving perceptual coherence while altering ontological status. Importantly, deception here does not rely on subtle distortion but on perfect perceptual fidelity to non‐existent referents.

This reliance on trust heuristics operates alongside deliberate cognitive engagement. Clinicians can be skeptical and can deliberately engage with clinical data. Skepticism and deliberate engagement are not alternatives to trust heuristics but operate within the background assumption that the input itself is authentic. A clinician can be highly skeptical about the interpretation of an image while still assuming that the image is a real image of the patient. The issue is not that clinicians never question or deliberate; it is that they cannot reasonably deliberate about every input's authenticity without verification systems. The paper does not claim that cognition relies exclusively on trust heuristics; it claims that trust heuristics are a necessary component of efficient clinical reasoning.

### 2.3 Time Pressure and Workload Constraints

Clinical decision‐making occurs under time constraints and cognitive load [6]. Verification procedures that are not embedded in workflows are therefore systematically bypassed—not due to negligence but due to structural infeasibility. The question is therefore not whether clinicians could verify inputs, but whether verification is structurally supported or systematically precluded.

### 2.4 The Illusion of Expertise Immunity

A common assumption is that clinical training confers immunity to deception. However, empirical work on diagnostic reasoning and automation bias suggests that expertise does not eliminate susceptibility to misleading inputs; it often increases confidence in interpretation of those inputs [7, 8]. The result is not epistemic failure at the level of reasoning alone, but at the level of input authenticity.

This vulnerability is not a claim about clinicians being incompetent or generally vulnerable. It is a claim about the structure of clinical epistemology: clinicians must trust the authenticity of inputs to do their work, and that trust can be exploited. Vulnerability here is vulnerability to *deception*, not vulnerability to error in general.

*Section summary*: Taken together, epistemic dependence, background trust, workflow constraints, and the limits of expertise explain why clinician vulnerability is structural rather than individual. Clinicians are not vulnerable because they are incompetent; they are vulnerable because the structure of clinical reasoning creates dependencies that can be exploited when the information environment becomes adversarial. This structural vulnerability is the foundation for the harms discussed in the following section.

### 2.5 Anchoring Vignette: The Synthetic Nodule

Before examining the three harms systematically, consider a plausible near‐future scenario. Dr. Chen, a community hospital radiologist, reviews a chest X‐ray ordered for a 58‐year‐old patient with cough and dyspnea. The image shows a 12 mm pulmonary nodule—subtle but characteristic of early malignancy. Dr. Chen documents the finding, recommends CT follow‐up, and alerts the referring physician. The patient undergoes biopsy, which carries a 5% pneumothorax risk. The biopsy is negative. The nodule is not there.

Weeks later, a cybersecurity review reveals that the hospital's PACS was breached, and 47 synthetic images were injected across three departments. Dr. Chen interpreted six of them. The nodule she saw was a pixel‐perfect fabrication. The patient experienced unnecessary procedural risk. Dr. Chen now questions every image she reads.

This vignette is not presented as evidence of current prevalence. It is a plausibility demonstration—a hypothetical scenario that follows from established technical capabilities [1] and documented system vulnerabilities [9]. The purpose is to make the abstract conceptual claims concrete, not to assert that this has occurred.

## 3 Three Harms of Clinician‐Facing Deepfakes

### 3.1 Why Synthetic Deception Is Ethically Distinct

Before examining the three harms, it is necessary to address a foundational question: why is clinician‐facing deepfake deception ethically different from ordinary clinical error, misinformation, or patient deception? This question is the most persistent objection to the paper's argument.

The distinction can be understood through four dimensions:

**Table:** 

Dimension	Ordinary error	Misinformation	Patient deception	Clinician‐facing deepfake
Source	Accidental	External	Patient‐originated	Adversarial
Evidence Status	Authentic	False	False/Simulated	Fabricated with perceptual fidelity
Clinician Role	Misinterpreter	Filter	Recipient	Unaware conduit
Target of Attack	Reasoning	Belief	Patient trust	Epistemic environment itself

**Ordinary clinical error** involves misinterpretation of authentic evidence. The clinician reasons correctly from evidence that is real but may be subtle, ambiguous, or misinterpreted. The error is in reasoning, not in the evidence itself. Expertise can reduce these errors through training and experience.

**Misinformation** involves external false content that clinicians may encounter and filter. The clinician is a gatekeeper who can assess credibility. The patient may encounter misinformation directly, but the clinician serves as a corrective filter.

**Patient deception** (e.g., symptom fabrication for secondary gain) involves a patient actively deceiving a clinician. This is an interpersonal credibility assessment problem. The clinician can assess the patient's credibility through clinical examination, history, and observation. Expertise provides tools for detecting deception.

**Clinician‐facing deepfakes** are qualitatively different. They attack the authenticity of evidence itself, not the credibility of the person or the accuracy of reasoning. The clinician reasons correctly from evidence that is perceptually indistinguishable from authentic evidence but entirely fabricated. Expertise does not help because the deception occurs at the level of input authenticity, not reasoning accuracy. The target is not the clinician's judgment but the epistemic environment in which judgment occurs.

This is why clinician‐facing deepfakes constitute a new ethical category. Ordinary error is accidental and correctable. Misinformation is external and filterable. Patient deception is interpersonal and assessable. Synthetic deception is adversarial, input‐level, and designed specifically to defeat expertise. It undermines the epistemic conditions under which professional responsibility is exercised.

The harm to clinicians is not merely being deceived. It is the corruption of the epistemic conditions under which professional responsibility is exercised. When a clinician cannot distinguish authentic from synthetic inputs, their ability to fulfill their professional obligations is compromised at the foundational level of evidence acquisition. This is a qualitatively different harm from being lied to by a patient or making an error in reasoning.

### 3.2 Harm 1: Diagnostic Error Cascades

A diagnostic error cascade occurs when synthetic inputs generate incorrect clinical interpretations that propagate through downstream decision‐making.

For example, a synthetic radiological lesion may trigger biopsy, surveillance imaging, or therapeutic intervention, each introducing risk. The harm is therefore not confined to misinterpretation but extends through the entire care pathway.

Diagnostic error research demonstrates that downstream propagation is a central feature of clinical error systems [10, 11]. Synthetic inputs introduce a new upstream source of such cascades: fabricated evidence rather than misinterpreted evidence. Importantly, this differs from conventional diagnostic error in that the initial epistemic input is not partially incorrect but entirely non‐referential while remaining perceptually coherent.

### 3.3 Harm 2: Testimonial Contamination

Testimonial contamination refers to the unintentional transmission of synthetic information through clinical authority channels.

If clinicians incorporate synthetic imaging findings, fabricated research, or manipulated patient‐generated media into reasoning, they may transmit conclusions to patients as clinically authoritative. This creates a second‐order epistemic harm: patients are not exposed to the synthetic material directly but to its institutional amplification through trusted testimony.

*Mechanism*: Testimonial contamination operates through a four‐step causal chain: (1) a clinician unknowingly consumes synthetic information (image, research paper, patient‐generated media); (2) the clinician incorporates this information into their clinical reasoning without verification; (3) the clinician communicates this information to a patient as if it were authentic clinical knowledge; (4) the patient acts on this information with justified trust in the clinician's epistemic authority. The contamination is testimonial because it corrupts the clinician's role as a trusted intermediary between evidence and patient. Unlike ordinary misinformation, which the patient might encounter directly, testimonial contamination exploits the very channel—professional medical testimony—that patients rely on for correction.

This mechanism extends established concerns about misinformation [12], but differs in structure: the clinician is not merely a filter but an unaware conduit of synthetic epistemic content originating inside clinical systems themselves.

*Empirical anchor*: Thunström [13] demonstrated that a fabricated disease (Bixonimania) entered the peer‐reviewed literature after AI chatbots treated it as real. While that study focused on AI‐generated text, the mechanism applies to synthetic clinical research: a clinician who reads a synthetic paper cannot distinguish it from authentic research.

### 3.4 Harm 3: Institutional Epistemic Fragility

Institutional epistemic fragility refers to the vulnerability of healthcare systems whose policies, guidelines, and aggregated knowledge depend on unverified or synthetic inputs.

Unlike individual diagnostic errors, institutional fragility operates at scale. A single fabricated study or manipulated dataset, if integrated into synthesis processes, may propagate across guidelines and decision frameworks.

While current documented cases remain limited, cybersecurity research confirms that healthcare systems are already targets of data manipulation and intrusion [9]. Synthetic data injection represents a conceptual extension of these threats into epistemically realistic but ontologically fabricated content.

Crucially, this vulnerability is not evenly distributed. Resource‐limited systems are structurally more exposed due to reduced verification infrastructure. This creates a distributional epistemic risk, not merely a technical one. Any adequate response framework must therefore include technology transfer, open‐source verification tools, and global governance mechanisms—not merely institutional recommendations for resourced settings.

*Summary*: Clinician‐facing deepfakes generate three distinct harms—diagnostic error cascades (unwitting intervention based on fabricated evidence), testimonial contamination (contamination of the clinician‐patient testimonial channel), and institutional epistemic fragility (systems‐level vulnerability to synthetic research and policy information). These harms are not reducible to patient‐facing harms and are not merely speculative: they follow from demonstrated technical capabilities and well‐documented vulnerabilities in clinical workflows.

## 4 Epistemic Role Reversal

### 4.1 Definition

We define **epistemic role reversal** as:

A condition in which the epistemic advantage typically associated with expertise is locally suspended due to adversarial manipulation of perceptual or testimonial inputs, such that expert judgment becomes structurally equivalent to lay perception in detecting authenticity.

This is not general expert fallibility. It is a domain‐specific collapse of reliability under adversarial input conditions.

Importantly, epistemic role reversal is not simply a description of ordinary expert fallibility. Ordinary expert fallibility involves probabilistic error within a trustworthy environment—the radiologist who occasionally misses a subtle finding on an authentic image. Epistemic role reversal describes a qualitatively different phenomenon: the systematic decoupling of perceptual reliability from epistemic justification under adversarial input conditions. In ordinary fallibility, the expert's error is a departure from reliable performance. In epistemic role reversal, the expert's error is the expected outcome of a perceptual system being fed inputs it was never designed to evaluate. The radiologist who misses a subtle real nodule and the radiologist who ‘sees’ a non‐existent nodule on a synthetic image are not making the same kind of mistake. The first is a stochastic performance failure; the second is a systematic vulnerability exploited by an adversary.

This concept does not claim that clinicians are equivalent to patients in all respects. It claims that deepfakes create specific conditions under which the clinician's epistemic advantage collapses—specifically, conditions of adversarial perceptual manipulation where the reliability of the information environment can no longer be assumed.

### 4.2 Distinction From Existing Concepts

Epistemic role reversal differs from:

- *Automation bias*: reliance on system outputs despite conflict with judgment.
- *Diagnostic error*: misinterpretation of authentic clinical data.
- *Misinformation exposure*: external false content in non‐clinical channels.
- *Epistemic dependence*: reliance on testimony in trustworthy systems.

The key distinction is that epistemic role reversal involves loss of access to authenticity discrimination at the input level, not merely reasoning error or weighting error. A clinician is not misinterpreting evidence; they are reasoning correctly from evidence that lacks stable ontological grounding.

In summary, epistemic role reversal is not a relabeling of known phenomena. It identifies a qualitatively distinct threat: the adversarial manipulation of perceptual heuristics in environments where trust has historically been rational. Recognizing this distinctness is necessary because the responses to automation bias (better calibration), diagnostic error (cognitive debiasing), misinformation (patient education), and epistemic dependence (trust) are all insufficient responses to adversarial perceptual collapse.

## 5 Clinical Epistemic Hygiene: A Precautionary Framework

If clinicians are vulnerable to deepfake deception, what should be done? This section proposes a framework for clinician‐facing verification—practices that clinicians and health systems can adopt to verify the authenticity of information they receive. Given the current uncertainty about real‐world prevalence, this framework is offered as precautionary: the potential harms are sufficiently severe to warrant proactive capability‐building, even before widespread incidents occur.

### 5.1 Normative Status of the Proposal

This framework does not introduce new foundational clinical duties. Existing obligations—accuracy, non‐maleficence, disclosure of error—remain unchanged.

What changes is the **feasibility condition** for fulfilling those duties under adversarial information conditions. Historically, clinicians could reasonably assume that images in PACS were authentic. That assumption can no longer be treated as universally warranted. But ‘be more careful’ is not an adequate response when images are perceptually indistinguishable. The only way to fulfill existing duties in a synthetic media environment is to build verification systems. The primary burden therefore shifts from individual clinicians to institutional systems capable of verifying epistemic provenance.

### Three levels of obligation

- **Individual clinicians** have the obligation to (a) recognize their vulnerability, (b) use available verification tools, and (c) disclose suspected synthetic incidents. These are not new duties but extensions of existing professional competence obligations.
- **Health systems** have the obligation to (a) deploy provenance verification infrastructure, (b) integrate verification into workflows, and (c) establish reporting systems. These are new obligations because the threat is new.
- **Professional bodies** have the obligation to (a) update standards of care to include verification, (b) develop training, and (c) advocate for technical standards (e.g., C2PA) that make verification possible.

Failure to distinguish these levels risks either demanding the impossible of individual clinicians (verifying every image unaided) or letting institutions off the hook (blaming clinicians for systematic failures).

### 5.2 Image and Data Provenance Verification

The recommendations that follow are not presented as immediately implementable standards applicable to all health systems today. Rather, they are offered as directionally necessary components of future clinical information architecture under conditions where synthetic media become prevalent. The timeline for implementation will vary by setting, and the specific technical solutions will evolve. What matters for present purposes is the direction of change—away from default trust in perceptual input and toward systematic verification—not the precise specifications.

Health systems should progressively adopt provenance verification systems for diagnostic imaging and digitally generated clinical data, including:

- Cryptographic signing at point of acquisition [14].
- Embedded verification in clinical viewing systems.
- Explicit flagging of unverifiable inputs.

It is important to acknowledge that technical tools will reduce much of the risk. Cryptography, watermarking, and metadata verification can detect many synthetic inputs. However, ethics concerns persist even after technical detection. A clinician whose judgment has been compromised by synthetic information may not know to verify it. Even if detected after the fact, the clinician has already been deceived, their judgment has been undermined, and the trust relationship has been violated. Technical solutions are necessary but insufficient; they must be complemented by institutional and professional norms.

### 5.3 Risk‐Based Verification Triggers

Verification should be selectively applied based on epistemic risk, including:

- Rare findings in low‐pretest‐probability contexts.
- Discordance between imaging and clinical presentation.
- High‐impact intervention pathways.

This avoids unrealistic expectations of universal verification.

### 5.4 Patient‐Generated Media

Patient‐provided audiovisual data should be treated as clinically informative but not inherently authenticated. Verification may include corroboration, metadata inspection where feasible, and clinical examination as primary validation.

### 5.5 Research Literature Authentication

The Thunström [13] study demonstrates that synthetic research can enter the literature. Clinicians and institutions should prioritize research from journals with robust provenance standards, use tools to check for fabricated studies, and treat unusual or surprising findings with increased skepticism pending verification.

### 5.6 Error Disclosure Protocols

When clinician deception is discovered—a radiologist learns that an image they interpreted was synthetic—disclosure to the patient is an ethical obligation. Protocols should include informing the patient that the diagnosis or recommendation was based on synthetic information, offering appropriate follow‐up, and documenting the error and system improvements.

### 5.7 Institutional and Global Constraints

Verification infrastructure is unevenly available globally. This is not a peripheral issue but a structural constraint. Resource‐limited systems are structurally more exposed due to reduced verification infrastructure. This asymmetry should be understood as a constitutive feature of the threat, not an ancillary limitation.

Accordingly, implementation requires:

- Open‐source verification tools.
- Low‐resource verification protocols (e.g., lightweight cryptographic hashing without real‐time validation).
- International standards coordination (e.g., WHO‐level guidance).
- Staged adoption models rather than uniform requirements.

*Summary*: Clinical epistemic hygiene comprises institutional verification infrastructure (provenance signing, verification triggers, disclosure protocols) and individual practices (skepticism toward unverified media, research authentication). The framework is precautionary rather than reactive, and its recommendations are directional rather than prescriptive. The three‐level obligation structure recognizes that individual vigilance is impossible without institutional support, and global asymmetry is a design constraint rather than an afterthought.

## 6 Virtues for Clinicians in Adversarial Epistemic Environments

Rather than expanding virtue lists, we identify three integrated epistemic dispositions. These are not presented as independent traits but as analytically separable dimensions of a single epistemic stance under adversarial uncertainty. A clinician who lacks any one dimension is vulnerable in ways that the others cannot fully compensate.

### 6.1 Epistemic Humility Under Adversarial Conditions

Recognition that perceptual coherence is no longer sufficient evidence of authenticity in some clinical domains. This virtue merges three related dispositions: recognizing that training does not confer immunity to deception, approaching clinical data as potentially synthetic without becoming paralyzed by skepticism, and maintaining meta‐cognitive awareness that confidence is not a reliable indicator of accuracy.

*Behavioral markers*: The epistemically humble clinician (a) accepts that synthetic images are perceptually indistinguishable from authentic ones, (b) does not rely on ‘clinical intuition’ to spot deepfakes, (c) actively seeks verification tools rather than trusting unaided perception, and (d) holds clinical judgments with appropriate openness to revision. For example, the radiologist who encounters a rare finding in a low‐risk patient should treat this as a verification trigger, not merely an interesting presentation.

### 6.2 Institutional Epistemic Vigilance

Commitment to embedding verification into workflow systems rather than relying on individual detection ability, coupled with the recognition that individual vigilance is insufficient without institutional support. Whereas epistemic humility says ‘I might be wrong,’ institutional epistemic vigilance says ‘I will build and use systems to check.’

*Behavioral markers*: The institutionally vigilant clinician (a) follows provenance verification protocols, (b) uses decision support triggers for suspicious findings, (c) documents information sources with sufficient detail to enable retrospective verification, (d) participates in institutional verification infrastructure design, and (e) advocates for verification tools at the departmental and hospital level. For example, the clinician who discovers a suspected synthetic image should report it through institutional safety systems rather than dismissing it as a one‐off anomaly.

### 6.3 Testimonial Integrity

Obligation to maintain provenance awareness in clinical communication and to disclose synthetic contamination when identified. This virtue follows directly from testimonial contamination as a harm: if clinicians are vulnerable to unknowingly transmitting fabricated information, they have a corollary duty to maintain the integrity of the testimonial channel. Testimonial integrity extends ordinary informed consent: patients have a right to know not only what the clinician recommends but also the provenance of the evidence on which that recommendation is based.

*Behavioral markers*: The clinician with testimonial integrity (a) when possible, shares information provenance with patients (“this recommendation is based on a study from Journal X that uses verified data”); (b) when synthetic deception is discovered, discloses this to affected patients even in the absence of clear harm, preserving the integrity of the testimonial channel; (c) reports suspected synthetic incidents to institutional safety systems; and (d) resists the temptation to conceal or minimize deception‐related errors.

### 6.4 Summary of Virtues

**Table:** 

Virtue	Core definition	Behavioral markers
Epistemic humility Under adversarial conditions	Recognition that perceptual coherence is not sufficient evidence of authenticity	Seeks verification tools; treats rare findings as triggers; accepts confidence ≠ accuracy
Institutional epistemic vigilance	Commitment to embedding verification into workflow systems	Follows protocols; reports incidents; advocates for infrastructure
Testimonial integrity	Maintaining provenance awareness and disclosing contamination	Shares provenance; discloses errors; resists concealment

## 7 Objections and Responses

### 7.1 Objection 1: This Is Speculative

*Response:* The argument is not that clinician‐facing deepfakes are currently prevalent, but that adversarial capability, demonstrated perceptual indistinguishability, and existing system vulnerabilities jointly justify precautionary preparation. The goal is not to predict the future but to provide ethical frameworks that can guide response if and when these technologies are used deceptively. Precautionary ethics does not require documented harm at scale; it requires plausible mechanisms for harm and sufficient severity to warrant advance preparation.

### 7.2 Objection 2: Verification Is Impractical

*Response:* The framework explicitly distinguishes between universal verification (not required) and risk‐triggered, system‐level verification (feasible). The claim is not that every image must be verified, but that systems should be capable of verification when epistemic risk warrants it.

### 7.3 Objection 3: Existing AI Safety and Cybersecurity Tools Suffice

*Response:* Technical tools may detect some synthetic inputs, but they cannot address the ethical and trust implications. Even if a synthetic input is detected after the fact, the clinician has already been deceived, their judgment has been undermined, and the trust relationship has been violated. Existing tools address system integrity and data security, not perceptual authenticity within clinical reasoning pipelines. Technical solutions are necessary but insufficient; they must be complemented by institutional and professional norms. Moreover, technical detection is reactive—it identifies deception after it has already occurred. The ethical harm of deception does not disappear once the deception is detected.

### 7.4 Objection 4: Harms Are Unproven

*Response:* The harms are structurally derived from established mechanisms of diagnostic error propagation and epistemic dependency under unreliable inputs. The absence of documented prevalence is not evidence of absence of harm potential. The burden is to show that the harms are plausible and severe enough to warrant precaution—not that they have already occurred.

### 7.5 Objection 5: Low‐Resource Settings Are Excluded

*Response:* Resource asymmetry is treated as a core design constraint requiring differentiated implementation pathways, not as an afterthought. The framework explicitly acknowledges that resource‐limited systems are structurally more exposed and requires open‐source tools, low‐resource verification protocols, and staged adoption models.

### 7.6 Objection 6: Clinicians Are Trained Professionals. Patients Are Not. The Asymmetry Is Justified

*Response:* The asymmetry is justified under ordinary epistemic conditions. Deepfakes create conditions where training does not help. Tordjman et al. [1] tested exactly this proposition: experienced radiologists with specialized training could not reliably detect synthetic images. The justification for asymmetry depends on the assumption that training provides epistemic advantage. When that assumption fails, the asymmetry collapses. The concern is not about knowledge asymmetry in general, but about asymmetric vulnerability to a specific type of deception that training cannot address.

## 8 Limitations

This paper is conceptual and does not estimate prevalence of clinician‐facing deepfakes in clinical systems. It establishes conceptual possibility, structural vulnerability, and normative implications.

The paper focuses on deepfakes where clinicians are the direct target of synthetic media. It does not fully address cases where patients use deepfakes to deceive clinicians (e.g., synthetic symptom videos for secondary gain) or where deepfakes target administrative decisions (insurance, licensing, credentialing). Each of these raises distinct ethical questions.

The framework is primarily developed with high‐resource health systems in mind, although scalability constraints and global asymmetry are explicitly addressed as constitutive features of the problem.

Empirical validation of epistemic role reversal, including measurement of detection failure rates under adversarial conditions, remains an open research area. Whether the proposed virtues can be taught, measured, or reliably practiced is likewise a subject for future research.

## 9 Conclusion

Clinical ethics has largely conceptualized deepfakes as a patient‐facing epistemic threat. This paper demonstrates that clinicians are equally embedded within systems vulnerable to synthetic deception.

Clinician‐facing deepfakes generate diagnostic error cascades, testimonial contamination, and institutional epistemic fragility. These are not merely symmetrical inversions of patient harms but structurally distinct failures of epistemic grounding in clinical systems.

We introduced the concept of epistemic role reversal to describe the collapse of perceptual epistemic advantage under adversarial conditions—the systematic decoupling of perceptual reliability from epistemic justification under adversarially generated clinical inputs. This phenomenon is not reducible to automation bias, diagnostic error, or misinformation, but represents a distinct class of epistemic vulnerability.

We proposed clinical epistemic hygiene as a precautionary framework combining provenance verification, risk‐based workflow design, and institutional epistemic safeguards, organized across three levels of obligation (individual, system, professional body). We also articulated three integrated epistemic dispositions—epistemic humility under adversarial conditions, institutional epistemic vigilance, and testimonial integrity—as analytically separable dimensions of a single epistemic stance under adversarial uncertainty.

Both patients and clinicians operate within increasingly synthetic epistemic environments. Clinical ethics must therefore expand its focus from protecting one side of the stethoscope to securing the integrity of the epistemic system that connects both.

## Funding

The author has nothing to report.

## Ethics Statement

The author has nothing to report.

## Consent

The author has nothing to report.

## Conflicts of Interest

The author declares no conflicts of interest.

## Data Availability

Data sharing is not applicable to this article as no datasets were generated or analysed during the current study.
